# Supplementary material for: Effects of Ultrasound-Assisted Immersion Freezing on the Protein Structure, Physicochemical Properties and Muscle Quality of the Bay Scallop (Argopecten irradians) during Frozen Storage
Source: Foods. 2022 Oct 18;11(20):3247. doi: 10.3390/foods11203247 (PMC9601341; doi:10.3390/foods11203247)
Supplement: Supplementary file 1 [file foods-11-03247-s001.zip › Table S1.pdf]

Table S1. Quantitative value of myosin heavy chain (MHC) and actin of myofibrillar proteins from the control adductor muscle of scallop (AMS) and 90-day frozen stored AMS with different treatments.

	MHC	Actin
Control	154 ± 0.5^{f}	148 ± 1.5^{e}
AF	166 ± 1.8^{d}	162 ± 1.2^{c}
IF	163 ± 0.5^{e}	155 ± 1.2^{d}
UIF-100	197 ± 0.4^{a}	186 ± 2.2^{a}
UIF-125	162 ± 0.9^{e}	148 ± 2.2^{e}
UIF-150	163 ± 0.9^{e}	145 ± 2.1^{e}
UIF-175	169 ± 0.7^{c}	157 ± 0.6^{d}
UIF-200	189 ± 0.3^{b}	177 ± 2.0^{b}

Data are expressed as mean ± standard deviation, mean values in a column with different letters (a–f) are significantly different.
