# Supplementary material for: Effects of Ultrasound-Assisted Immersion Freezing on the Protein Structure, Physicochemical Properties and Muscle Quality of the Bay Scallop (Argopecten irradians) during Frozen Storage
Source: Foods. 2022 Oct 18;11(20):3247. doi: 10.3390/foods11203247 (PMC9601341; doi:10.3390/foods11203247)
Supplement: Supplementary file 1 [file foods-11-03247-s001.zip › Table S2.pdf]

Table S2. T_{max} (°C) and denaturation enthalpy ΔH (J/g) corresponding to DSC thermograms of control adductor muscle of scallop (AMS) and 90-day frozen stored AMS with different treatments.

	T_{max1} (°C)	T_{max2} (°C)	ΔH_1 (J/g)	ΔH_2 (J/g)
Control	48.19 ± 0.04^{a}	72.26 ± 0.10^{a}	0.84 ± 0.01^{a}	0.45 ± 0.02^{a}
AF	45.83 ± 0.08^{d}	69.07 ± 0.07^{d}	0.53 ± 0.02^{de}	0.35± 0.02^{b}
IF	46.16 ± 0.07^{cd}	68.22 ± 0.09^{e}	0.58 ± 0.03^{bcd}	0.37 ± 0.03^{b}
UIF-100	47.16 ± 0.14^{b}	67.10 ± 0.12^{f}	0.62 ± 0.02^{b}	0.34 ± 0.01^{b}
UIF-125	46.23 ± 0.06^{c}	70.19 ± 0.04^{c}	0.57 ± 0.04^{bcd}	0.38 ± 0.01^{ab}
UIF-150	46.32 ± 0.11^{bc}	71.05 ± 0.06^{b}	0.63 ± 0.02^{b}	0.39± 0.04^{ab}
UIF-175	45.81 ± 0.13^{d}	70.25 ± 0.07^{c}	0.55 ± 0.02^{de}	0.36 ± 0.03^{b}
UIF-200	46.19 ± 0.08^{c}	67.29 ± 0.08^{f}	0.60 ± 0.02^{bc}	0.35 ± 0.02^{b}

Data are expressed as mean ± standard deviation, mean values in a column with different letters (a–f) are significantly different.
