# Supplementary material for: Effects of Ultrasound-Assisted Immersion Freezing on the Protein Structure, Physicochemical Properties and Muscle Quality of the Bay Scallop (Argopecten irradians) during Frozen Storage
Source: Foods. 2022 Oct 18;11(20):3247. doi: 10.3390/foods11203247 (PMC9601341; doi:10.3390/foods11203247)
Supplement: Supplementary file 1 [file foods-11-03247-s001.zip › Table S3.pdf]

Table S3. Comprehensive score of control adductor muscle of scallop (AMS) and 90-day frozen stored AMS with different treatments.

Variables	PC1 (59.52%)	PC2 (28.82%)
$\triangle H_1$	0.973	-0.032
α-helix	0.928	-0.364
T_{max1}	0.923	0.17
Thawing loss	-0.923	0.323
TVB-N	-0.921	0.385
Random coil	-0.878	0.165
Hardness	0.873	-0.441
Chewiness	0.868	-0.271
$\triangle H_2$	0.824	-0.553
TBARS	-0.824	0.175
Springiness	0.819	-0.519
MHC	-0.262	0.911
Actin	-0.261	0.891
T_{max2}	0.526	-0.811
β-turn	-0.581	0.761
β-sheet	-0.314	-0.659
